# The Mouse *Levator Auris Longus* Muscle: An Amenable Model System to Study the Role of Postsynaptic Proteins to the Maintenance and Regeneration of the Neuromuscular Synapse

**DOI:** 10.3389/fncel.2020.00225

**Published:** 2020-07-29

**Authors:** Jorge Ojeda, Francisca Bermedo-García, Viviana Pérez, Jessica Mella, Patricia Hanna, Daniel Herzberg, Rocío Tejero, Mario López-Manzaneda, Lucia Tabares, Juan Pablo Henríquez

**Affiliations:** ^1^Neuromuscular Studies Laboratory (NeSt Lab), Department of Cell Biology, Faculty of Biological Sciences, Center for Advanced Microscopy (CMA BioBio), Universidad de Concepción, Concepción, Chile; ^2^Department of Medical Physiology and Biophysics, School of Medicine, Universidad de Sevilla, Sevilla, Spain; ^3^Developmental Neurobiology Unit, Biomedical Sciences Research Laboratory, Basic Sciences Department, Faculty of Medicine, Universidad Católica de la Santísima Concepción, Concepción, Chile; ^4^Veterinary Sciences Clinic, Universidad de Concepción, Concepción, Chile

**Keywords:** neuromuscular junction, presynaptic, postsynaptic, regeneration, electroporation, skeletal muscle

## Abstract

The neuromuscular junction (NMJ) is the peripheral synapse that controls the coordinated movement of many organisms. The NMJ is also an archetypical model to study synaptic morphology and function. As the NMJ is the primary target of neuromuscular diseases and traumatic injuries, the establishment of suitable models to study the contribution of specific postsynaptic muscle-derived proteins on NMJ maintenance and regeneration is a permanent need. Considering the unique experimental advantages of the *levator auris longus* (LAL) muscle, here we present a method allowing for efficient electroporation-mediated gene transfer and subsequent detailed studies of the morphology and function of the NMJ and muscle fibers. Also, we have standardized efficient facial nerve injury protocols to analyze LAL muscle NMJ degeneration and regeneration. Our results show that the expression of a control fluorescent protein does not alter either the muscle structural organization, the apposition of the pre- and post-synaptic domains, or the functional neurotransmission parameters of the LAL muscle NMJs; in turn, the overexpression of MuSK, a major regulator of postsynaptic assembly, induces the formation of ectopic acetylcholine receptor clusters. Our NMJ denervation experiments showed complete reinnervation of LAL muscle NMJs four weeks after facial nerve injury. Together, these experimental strategies in the LAL muscle constitute effective methods to combine protein expression with accurate analyses at the levels of structure, function, and regeneration of the NMJ.

## Introduction

The vertebrate neuromuscular junction (NMJ) is a peripheral cholinergic synapse formed by a motor axon terminal, a specialized acetylcholine receptor (AChR)-enriched fraction of the muscle membrane, and terminal Schwann cells. The NMJ displays a high degree of subcellular specialization, large size, and easy experimental access, features that have significantly contributed to uncovering the principles of synaptic formation, growth, maturation, and maintenance *in vivo* (Sanes and Lichtman, 2001). Indeed, the ultrastructure of synaptic architecture and the principles of synaptic transmission were first characterized at the frog NMJ (Birks et al., 1960; Katz and Miledi, 1969; Katz, 1971).

To achieve its mature, complex shape, the NMJ undergoes drastic modifications during early postnatal development. At the muscle membrane, initial small “plaque”-like uniform AChR densities are sequentially transformed into bigger elaborate branches with a “pretzel”-like shape (Sanes and Lichtman, 2001; Shi et al., 2012). These morphological changes are closely associated with NMJ function. For instance, immature NMJs have lower amplitude evoked endplate potentials (EPPs), lower quantal content, and higher latency than mature NMJs (Bewick et al., 2004; Cano et al., 2013).

Severe motor pathologies and traumatic nerve injuries cause NMJ dysfunction (Moloney et al., 2014; Ko and Robitaille, 2015; Martineau et al., 2018). Upon NMJ denervation, the postsynaptic apparatus displays a remarkable maintenance ability, which, within a discrete time frame, allows successful functional regeneration (Sakuma et al., 2016). Even though muscle proteins, including local intracellular effectors and extracellular components, are thought to regulate the stability of AChR clusters after denervation (Bloch-Gallego, 2015), the identity and contribution of muscle-derived molecular mechanisms helping the maintenance of postsynaptic structures are still to be fully elucidated.

The *levator auris longus* (LAL) muscle offers unique experimental advantages to study the neuromuscular synapse. It is a superficially exposed muscle, which facilitates genetic modulation of post- and trans-synaptic proteins expression, drug delivery approaches, and *in vivo* time-lapse imaging to reinvestigate the same NMJs over time. The LAL is a mainly fast-twitch muscle that functions to move the pinna. It is constituted by flat and thin rostral and caudal portions, each having two to three muscle fiber layers, localized in the dorsal surface of the skull (Erzen et al., 2000; Murray et al., 2010a; Wright et al., 2011). It is innervated by a posterior auricular branch of the facial nerve, thus generating a well-described pattern of five different rostrals (R1–R5) and two caudal (C1–C2) innervation zones (Murray et al., 2008, 2010b). Together, these features have facilitated NMJ morphological studies (Angaut-Petit et al., 1987; Murray et al., 2008; Klooster et al., 2012). The LAL muscle has also been extensively employed for electrophysiological recording in *ex vivo* nerve/muscle preparations (Katz et al., 1996; Ruiz et al., 2010; Burke et al., 2018).

In this work, we extend the convenience of the LAL muscle through efficient electroporation-mediated gene transfer and integrative morpho-functional analysis of the NMJ. Also, we have standardized nerve injury protocols to analyze NMJ regeneration. Together, these experimental strategies represent accessible *in vivo* screening methods to analyze the contribution of muscle proteins on NMJ morphology, function and regeneration.

## Materials and Methods

### Animals

Experimental procedures were approved by the Bioethics Committee at Universidad de Concepción, Chile, and followed the norms imposed by the Bioethics Committee of the National Commission for Scientific and Technological Research, Chile (CONICYT), as well as the guidelines of the European Council Directive for the Care of Laboratory Animals. Experimental procedures were conducted in P21 or adult male mice under sedation (2.5% Isofluorane with a 0.8–1 l/min oxygen mixture). Before LAL muscle dissection, animals were euthanized by an overdose of isofluorane or carbon dioxide.

### *In vivo* Muscle Electroporation

LAL muscles from P21 or adult mice were electroporated following a method described for limb muscles, with minor modifications (DiFranco et al., 2009). The control DNA used through these studies was tdTomato-N1 (a gift from Michael Davidson, Nathan Shaner, and Roger Tsien; Addgene plasmid # 54642). To induce MuSK overexpression, we used the pBK-CMV-Δlac-rMuSK plasmid, which contains the exact coding sequence of rat MuSK (GenBank sequence accession number U34985, except for the 10 amino acid insert in the first splice site between E209 and V210; a kind gift of Dr. Jonathan B. Cohen, Harvard Medical School, MA, USA) fused to the myc tag sequence (rMuSK-myc; Bianchetta et al., 2005). We also used the MuSK-EGFP plasmid, which contains the full-length mouse MuSK coding sequence (a kind gift of Dr. William D. Phillips, University of Sydney, Camperdown, NSW, Australia; Ghazanfari et al., 2015). Plasmid DNAs were purified according to the Qiagen Maxiprep protocol following the instructions of the manufacturer. To facilitate the DNA plasmid access to the muscle surface, 10 μl of a 2 mg/ml solution of hyaluronidase (ref. H3884, Sigma-Aldrich) were applied under sedation by a subcutaneous injection using a Hamilton syringe (McMahon et al., 2001). After 1 h, mice were re-anesthetized and a 5–10 mm surgical skin incision was performed at the level of skull sagittal suture to expose the LAL muscles. Then, different amounts of total plasmid DNA coding for tdTomato or EGFP (controls), or rMuSK-myc or MuSK-EGFP in a final volume of 10 μl in 0.01 M PBS were injected just underneath the muscle fascia forming a bubble. The pBK-CMV-Δlac-rMuSK plasmid was co-electroporated along with the tdTomato-N1 plasmid in a 5:1 ratio. For the DNA electrotransfer procedure, two gold needle-type electrodes (Genetrodes, BTX Harvard Apparatus, Holliston, MA, USA) separated by 5 mm were positioned on the entire transversal length of the LAL muscle to deliver five pulses of 100 V/cm of 20 ms duration at 1 Hz using an ECM 830 electroporator (BTX Harvard Apparatus). This procedure was repeated in the contralateral Hemi-LAL muscle. Finally, the skin was sutured using absorbable monofilament surgical suture (Ethicon Vicryl USP 6–0) and animals were monitored until their recovery.

### Facial Nerve Injury

Facial nerve injuries were performed as described (Olmstead et al., 2015) with some modifications. Briefly, adult animals were anesthetized by isofluorane inhalation, as described above, and after shaving the right ear posterior region, a surgical 5 mm skin incision was performed to expose the facial nerve branches. The most dorsal branch of the facial nerve innervating the LAL muscles was carefully cleared avoiding direct manipulation. In the NMJ degeneration protocol, a section of 4 mm of the facial nerve branch was transected; in turn, for the reinnervation protocol, the facial nerve branch was crushed for 30 s using Dumont #5/45 forceps (Fine Science Tools). Control experiments only considered skin incision and facial nerve exposure. Finally, the skin was sutured using absorbable monofilament surgical suture (Ethicon Vicryl USP 6–0) and animals were monitored until their recovery.

### Muscle Histology Analyses

At the indicated times after electroporation, mice were euthanized and the LAL muscles were dissected, fixed in 0.5% formaldehyde in 0.01 M PBS at 22°C for 90 min and embedded in optimal cutting temperature (OCT) compound (Sakura Fine Technical Company, Torrance, CA, USA). Samples were sectioned every 20 μm with a cryostat (Thermo Scientific Microm HM 525) and mounted on Vectabond (Vector Laboratories, Burlingame, CA, USA) coated slides. Muscle fiber morphology was analyzed through conventional hematoxylin/chromotrope staining (Woehlbier et al., 2016). Cryosections were also stained with an NADH reduced solution (Tris-buffer, pH 7.4, NADH reduced, nitro-blue tetrazolium; Sigma Aldrich, St. Louis, MO, USA) for 45 min, and fibers were classified into oxidative (dark blue) or non-oxidative (light blue). The identity of all muscle fibers contained within the Hemi-LAL muscles and the cross-sectional area (CSA) of >100 fibers per type in each Hemi-LAL were determined using the ImageJ software. To reveal the muscle membrane and nuclei, cryosections were stained with the wheat germ agglutinin lectin (WGA, Molecular Probes, Waltham, MA, USA (1 μg/ml) conjugated to Alexa488) plus DAPI (1 μg/ml), respectively, during 10 min (Woehlbier et al., 2016).

### NMJ Staining, Imaging, and Analyses

Whole-mounted LAL muscles were fixed as described (Pérez-Garcia and Burden, 2012). After washing with 0.01 M PBS/0.5% v/v Triton X-100 for 2 h, samples were incubated with 0.1 M glycine in PBS for 30 min. Blocking was performed with 4% BSA dissolved in PBS/0.5% Triton X-100. Primary antibodies against neurofilaments (2H3, 1:300) plus synaptic vesicles (SV2, 1:200; both form the Developmental Studies Hybridoma Bank, DSHB, Department of Biology, University of Iowa, IA, USA) were incubated overnight in blocking solution (PBS/0.5% Triton X-100/4% BSA). After washing, samples were incubated with the secondary antibodies (Cy2 1:250; Donkey H + L, Jackson Immunoresearch Laboratories, West Grove, PA, USA) along with Alexa647- or Alexa488-conjugated α-bungarotoxin (αBTX, Molecular Probes; 1:500) overnight at 4°C, and subsequently mounted between two coverslips in DAKO fluorescence medium.

Images were acquired using an inverted Zeiss LSM 780 multiphoton or an LSM 700 laser scanning confocal microscope (CMA BioBio, Universidad de Concepción, Concepción, Chile). Confocal *z*-plane optical sections (1 μm) were captured using 25× (LD LCI Plan-Apochromat 25×/0.8 Imm Korr DIC M27), 40× (Plan-Apochromat 40×/1.3 Oil DIC M27), and 63× (Plan-Apochromat 63×/1.40 Oil DIC M27) objectives. Additionally, tilt-scan microscopy was employed to acquire the entire whole-mount LAL muscles. To adjust the fluorescence intensity in the deepest *z*-planes without varying the power of the laser scanning, the “auto *z* brightness correction” was used, which allows an automatic and linear interpolation of values amongst neighboring positions within the *z* stack. The electroporation efficiency was quantified as the ratio between fibers expressing tdTomato vs. the total fibers labeled with Alexa488-WGA. The postsynaptic morphometric analyses were performed as described (Bolliger et al., 2010; Jones et al., 2016; Woehlbier et al., 2016). At least 40–50 postsynaptic apparatuses from control or tdTomato-expressing fibers were counted per mice. To determine the NMJ innervation pattern, confocal images were analyzed as described (Jones et al., 2016). Briefly, the area of the pre-synaptic motor terminal within the NMJ region and the total AChR positive area of >45 NMJs per mice were calculated. Data are presented as the % of the apposition of postsynaptic AChR clusters by presynaptic motor axons.

### Electrophysiological Intracellular Recording

LAL muscle *ex vivo* preparations containing an intact 5 mm facial nerve stump were transferred to the stage of an Olympus BX50WI upright microscope and continuously perfused with an external solution (in mM: 135 NaCl, 5 KCl, 1 MgCl_2_, 12 NaHCO_3_, 12 glucose, and 2 CaCl_2_) at room temperature. First, the viability of the muscle preparation was evaluated by visual inspection under the microscope of muscle contraction after nerve stimulation at 2–15 V. Then, we evaluated action potentials in properly impaled muscle fibers having resting potentials between −50 and −70 mV. The recording electrode resistance was 15–25 MΩ. Evoked (EPP) and spontaneous miniature (mEPP) end-plate potentials were recorded and analyzed as described (Tejero et al., 2016). Briefly, the nerve was stimulated through square-wave pulses at the indicated frequencies using a suction electrode. A glass micropipette filled with 3 M KCl was connected to an intracellular recording amplifier (Neuro Data IR283, Cygnus technology, Southport, NC, USA) through a chloride silver wire and used to impale single muscle fibers near the motor nerve endings. Muscle contraction was prevented by including in the bath 3–4 mM μ-conotoxin GIIIB (Alomone Laboratories, Jerusalem, Israel), a specific blocker of muscular voltage-gated sodium channels. The data were analyzed as previously described (Tejero et al., 2016, 2020). EPP amplitudes were normalized to −70 mV and corrected for nonlinear summation. All electrophysiological data are expressed as group mean values ± SEM, with *n* and *N* being the number of NMJs and the number of mice, respectively. All reported results are based on 23–24 fibers from at least three animals per condition.

### Statistical Analyses

The statistical comparison was performed amongst data obtained from control and electroporated age-matched animals using Student’s unpaired *t*-test, based on a normal distribution of the data. In facial nerve injury experiments, quantification of parameters obtained from control and experimental animals at different times were compared using one-way ANOVA. Results were considered statistically significant when the *p*-value was < 0.05.

## Results

### Efficient Electroporation-Mediated Gene Transfer of LAL Muscles

**Figure 1:**
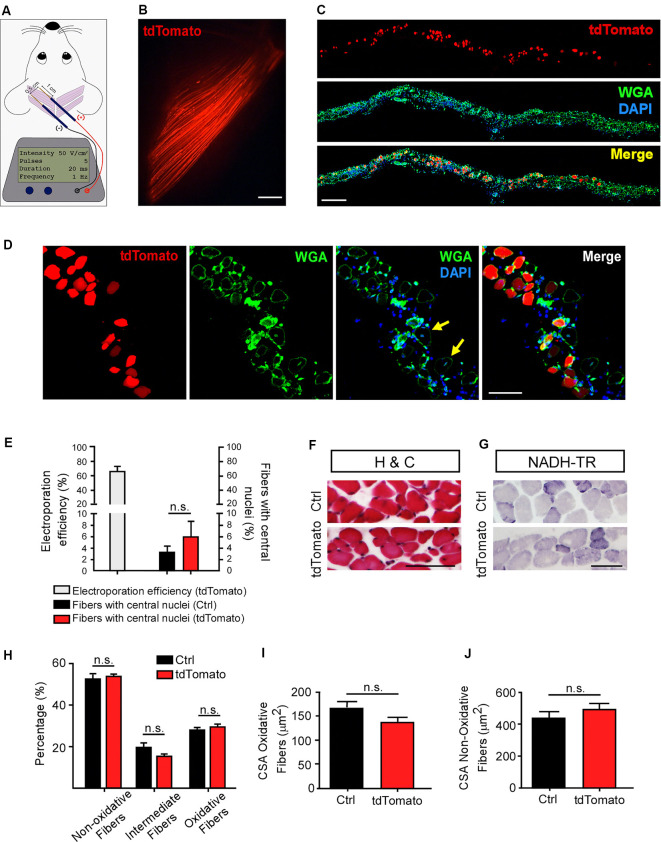
Efficient electroporation-mediated gene transfer of Levator auris longus (LAL) muscles. LAL muscles from adult mice were electroporated *in vivo* with a plasmid coding for the tdTomato protein. Plasmid DNA was injected underneath the LAL muscle fascia and gold needle-type electrodes were positioned above the muscle to deliver five pulses of 100 V/cm^2^ of 20 ms of duration at 1 Hz **(A)**. After 21 days, dissected whole-mounted muscles **(B)** were transversally sectioned and stained with Alexa488-WGA (green) and DAPI (blue) **(C)** to label the muscle membrane and nuclei, respectively. **(D)** Higher magnification images were used to quantify the efficiency of the procedure [as the percentage of tdTomato-expressing fibers from total fibers quantified based on wheat germ agglutinin (WGA) staining] **(E)** and the presence of central nuclei (arrowheads), as a parameter of muscle fiber damage/regeneration (E). **(F)** Transversal cryosections stained with Hematoxylin/Chromotrope revealed no significant alterations in muscle fiber histology or mononuclear cell infiltration. **(G)** NADH-TR histochemical activity detection was used to analyze non-oxidative (light and middle blue) and oxidative (dark blue) fibers. The proportion of these fiber types was quantified and expressed as a percentage of total fibers in the region of interest **(H)**. Also, the cross-sectional area (CSA) of oxidative **(I)** and non-oxidative **(J)** fibers was determined in >100 fibers per type in each Hemi-LAL. The results represent the mean ± SEM of N: three mice per group (control and electroporated). Scale bar 5 mm **(B)**, 200 μm **(C)**, 50 μm **(D)**, 50 μm **(F,G)**. *p* > 0.05, *t*-test; n.s.= non-significant.

The non-viral transfection by electroporation has proven to be an advantageous physical method to allow efficient gene transfer into muscle fibers *in vivo* (McMahon et al., 2001; Bloquel et al., 2004). Following this approach, gain and loss of function of specific genes have been employed to study skeletal muscle recovery in pathological contexts (Schertzer and Lynch, 2006; van der Pijl et al., 2016) and to address the contribution of muscle-derived proteins to the dynamic processes controlling NMJ morphology and function (Kong et al., 2004; Losen et al., 2005; Sadasivam et al., 2005; Punga et al., 2011; Chen et al., 2014; Wang et al., 2017). Based on this, we first standardized in LAL muscles an electroporation-mediated gene transfer protocol previously reported to be effective in the hind limb *flexor digitorum brevis* (FDB) muscle (DiFranco et al., 2009; Figure 1A). We chose to work with P21 mice as it coincides with the onset of plaque to the pretzel transition of the NMJ postsynaptic apparatus (Bolliger et al., 2010). A low magnification representative image reveals that a high proportion of rostral LAL muscle fibers express the tdTomato protein (Figure 1B). Transversal cryosections of the electroporated muscles were stained with the WGA lectin to reveal the sarcolemma, whereas nuclei were counterstained with DAPI (Figures 1C,D). This procedure allowed us to determine that the electroporation efficiency achieved an average of 66.7 ± 5.94% of total fibers (Figure 1E). It also allowed us to quantify the presence of central nuclei as a parameter of muscle regeneration after damage (Figure 1D). Quantification showed a similarly low percentage of fibers containing central nuclei in tdTomato-positive fibers (5.9 ± 2.7%) than in control non-electroporated fibers (3.2 ± 1.07%, non-significant, paired *t*-test; Figure 1E), revealing that the electroporation procedure did not result in muscle damage. To analyze if the electroporation procedure induced morphological defects in the LAL muscle, we performed histological analyses of LAL transversal cryosections. Our results show no gross differences in muscle fiber distribution or mononuclear cells infiltration (haematoxylin/chromotrope staining; Figure 1F). We also performed histochemical staining to reveal NADH-thioreductase activity to analyze potential changes in different muscle fiber-types after electroporation-mediated expression of tdTomato. Electroporated and control non-electroporated LAL muscles fibers are mostly fast-twitch (non-oxidative and intermediate; light and middle blue), whereas only a small proportion of fibers show an oxidative (dark blue) slow-twitch phenotype (Figures 1G,H). We did not find changes in the cross-sectional area (CSA) of both, oxidative and non-oxidative muscle fibers of electroporated compared to control LAL muscles (Figures 1I,J). Thus, our data show that an efficient electric field permeabilization procedure or the expression of a control protein does not affect LAL muscle morphology.

### The *in vivo* Electroporation Procedure in the LAL Muscle Does not Affect Neuromuscular Transmission

**Figure 2:**
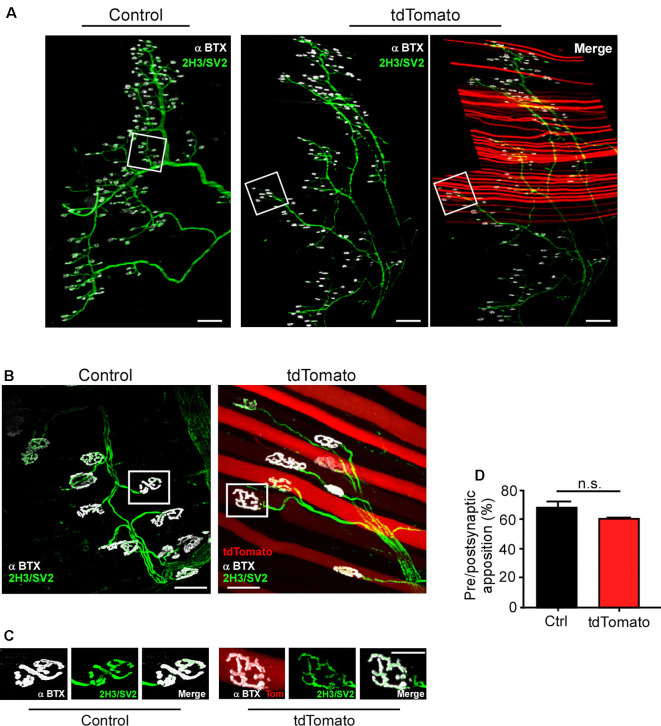
The *in vivo* electroporation procedure in the LAL muscle does not affect neuromuscular junction (NMJ) innervation. LAL muscles from control adult mice and those expressing the tdTomato protein for 21 days were dissected and subjected to immunohistochemistry with the 2H3 (neurofilament) plus SV2 (synaptic vesicles) antibodies to reveal presynaptic motor terminals, along with Alexa647-BTX (white pseudocolor) to stain postsynaptic densities. **(A)** Low magnification images of whole-mount preparations show that the NMJ profile from the R5 region of right Hemi LAL muscles is maintained in tdTomato-expressing fibers, as compared to controls. **(B,C)** Higher magnification confocal images show that terminal motor axon branches contact postsynaptic apparatuses in control and electroporated muscle fibers. **(D)** Quantification of the apposition of postsynaptic acetylcholine receptor (AChR) pretzels by presynaptic motor axons. The plots correspond to >45 NMJs per mice. The bars represent the mean ± SEM of N: three mice per group (control and electroporated). Scale bar 200 μm **(A)**, 5 μm **(B,C)**. *p* > 0.05, *t*-test; n.s.= non-significant.

**Figure 3:**
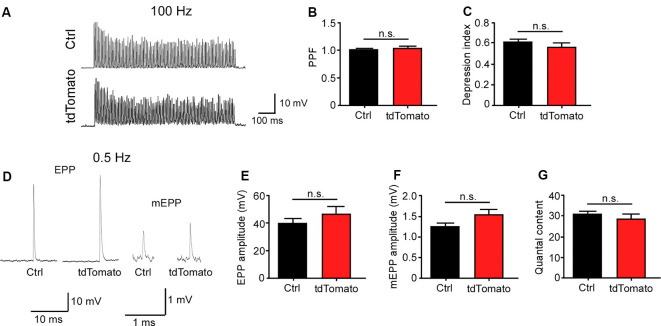
Neuromuscular transmission is not altered after electroporation-mediated gene transfer of the LAL muscle. For functional studies, LAL muscles overexpressing the tdTomato protein at P14 were analyzed through electrophysiological intracellular recording at P21. Non-electroporated LAL muscles from age-matched mice were used as controls. After blocking muscle contraction, stimulation trains of 100 Hz during 1 s **(A)** showed no changes in synaptic plasticity, evidenced by paired-pulse facilitation (PPF) **(B)** and depression index **(C)** quantification in control and electroporated fibers. Plots represent the average ± SEM of N: 3, n: 24 (control and electroporated fibers). **(D)** Representative end plate potential (EPP) and miniature EPP (mEPP) traces after 0.5 Hz stimuli of NMJs from control and tdTomato-expressing fibers. Amplitudes of EPPs **(E)**, mEPPs **(F)**, and the quantal content **(G)** of control and electroporated fibers were quantified. Plots represent the average ± SEM of N: 3, n: 24 (control and electroporated fibers). *p* > 0.05, *t*-test; n.s.= non-significant.

Next, we aimed to determine if the neuromuscular synaptic function could be compromised by our procedure. First, we analyzed the NMJ innervation profile (Figure 2A). Low magnification of the LAL R5 region shows no gross differences in the innervation pattern of tdTomato-expressing fibers, compared to controls (Figure 2A). Next, NMJs were evaluated for evidence of denervation based on whether the endplate marked by AChR staining lacked an overlying nerve terminal, visualized by immunohistochemical detection of neurofilament plus synaptic vesicles proteins (Figure 2B). In our experimental conditions, NMJs looked fully innervated, as nerve terminal branches aligned precisely with the postsynaptic specialization in both tdTomato-expressing and control non-electroporated muscles (Figures 2B,C). Quantitative analyses confirmed our observations, as presynaptic staining apposes more than 60% of the postsynaptic domain in both muscles, an expected quantification for fully innervated NMJs (Figure 2D; Jones et al., 2016). Second, we analyzed neuromuscular communication in *ex vivo* nerve/muscle LAL preparations using electrophysiological intracellular recording. We examined the characteristics of short-term synaptic facilitation and depression by stimulating the facial nerve at a high frequency (100 Hz) for 1 s (Figure 3A). Under these conditions, efficient recycling and replacement of the readily releasable pool of synaptic vesicles are required during the stimulus train (Ruiz et al., 2011). We found that paired-pulse facilitation (PPF) ratio (control: 1.02 ± 0.02; tdTomato: 1.03 ± 0.04; *p* > 0.05 *t*-test; Figure 3B) and the depression index (control: 0.60 ± 0.03; tdTomato: 0.56 ± 0.04; *p* > 0.05 *t*-test; Figure 3C) were unaffected by the electroporation protocol or by the expression of tdTomato. Also, a low-frequency stimulation protocol (0.5 Hz; Figure 3D) showed no significant differences in the amplitudes of evoked EPPs in LAL muscle fibers of electroporated animals (46.12 ± 5.6 mV) compared to control non-electroporated ones (39.41 ± 3.41 mV; *p* > 0.05 *t*-test; Figure 3E). Similarly, the average amplitude of spontaneous mEPPs in electroporated fibers (1.545 ± 0.12 mV) was similar to controls (1.252 ± 0.09 mV; *p* > 0.05 *t*-test; Figure 3F). As the amplitude of EPPs depends on both, the number of released quanta and postsynaptic proteins (Del Castillo and Katz, 1954), we also calculated the quantal content during a 5-min period of continuous recording of each nerve terminal (Figure 3G). Our results indicate that the electroporation procedure does not affect the number of quanta released per action potential with values of 31.05 ± 1.56 and 28.62 ± 2.58 (*p* > 0.05 *t*-test) for control and td-Tomato expressing muscle fibers, respectively. Together, these data evidence that the electroporation of LAL muscles to overexpress the red fluorescent protein tdTomato does not affect the neuromuscular transmission.

### Electroporation-Mediated Gene Transfer of tdTomato Does not Alter NMJ Post-synaptic Maturation

**Figure 4:**
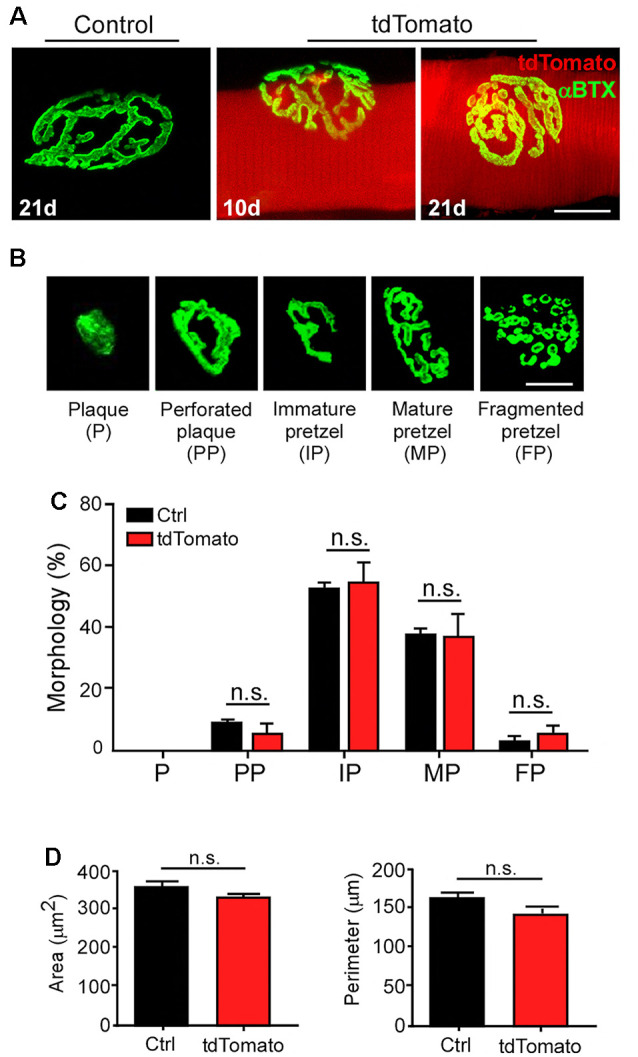
Electroporation-mediated gene transfer of tdTomato does not alter NMJ postsynaptic maturation. LAL muscles from control adult mice and those expressing the tdTomato protein for 10 and 21 days were dissected and stained with Alexa488-BTX. **(A)** AChR pretzels on the surface of tdTomato-expressing fibers were similar to those on control non-electroporated fibers **(A)**. To analyze NMJ maturation, electroporated LAL muscles at P21 were analyzed at P42. Non-electroporated age-matched mice were used as controls. AChR clusters were categorized into plaques (P), perforated plaques (PP), immature pretzels (IP), mature pretzels (MP), and fragmented pretzels (FP; **B**, *upper panel*) and their relative abundance were plotted **(B**, *lower panel***)**. Postsynaptic area **(C)** and perimeter **(D)** were also determined. Plots represent the average ± SEM of N: 3, n: 130 (Control), and N:3, n: 149 (tdTomato-expressing fibers). Scale bar 50 μm **(A)**, 25 μm **(B)**. *p* > 0.05, *t*-test; n.s.= non-significant.

Next, we aimed to analyze the morphology of postsynaptic domains after electroporation-mediated gene transfer. Similar pretzel-like postsynaptic morphologies were observed 10 and 21 days after muscle electroporation, compared to control non-electroporated fibers (Figure 4A). To quantitatively assess whether our procedure affects the NMJ structure, we analyzed the postsynaptic morphological transition occurring during NMJ maturation (Bolliger et al., 2010). As the most relevant morphological changes in the postsynaptic domain occur during early postnatal NMJ maturation (Sanes and Lichtman, 2001; Bolliger et al., 2010; Shi et al., 2012), the relative proportion of maturing postsynaptic shapes was quantified at P42, three weeks after electroporation. With that aim, AChR clusters were categorized into plaques (small uniformly distributed AChR clusters), perforated plaques (those containing internal poor-AChR regions), immature pretzels (exhibiting a peripheral opening), mature pretzels (complex highly branched shapes), and fragmented pretzels (more than four fragments; Figure 4B). As expected for P42 muscle fibers, we found no early plaque-like structures and a very small proportion of fragmented postsynaptic structures in control LAL fibers. Most structures corresponded to immature and mature pretzels (Figure 4B). Quantification shows that the electroporation protocol and the persistent expression of tdTomato did not alter the relative proportion of maturing NMJ structures, compared to control non-electroporated LAL muscles (Figure 4B). Also, quantification of other NMJ morphology parameters, such as the average area (tdTomato: 328.0 ± 11.13 μm^2^; control: 355.6 ± 15.50 μm^2^; *p* > 0.05 *t*-test; Figure 4C) or the perimeter (tdTomato: 141.7 ± 10.37 μm; control: 162.2 ± 7.99 μm; *p* > 0.05 *t*-test; Figure 4D) of postsynaptic apparatuses showed no differences, revealing that the maturation and stability of the NMJ postsynaptic domain are not affected by the electroporation procedure or by the expression of a control fluorescent protein.

### Electroporation-Mediated Overexpression of the MuSK Receptor Results in Ectopic AChR Clustering

**Figure 5:**
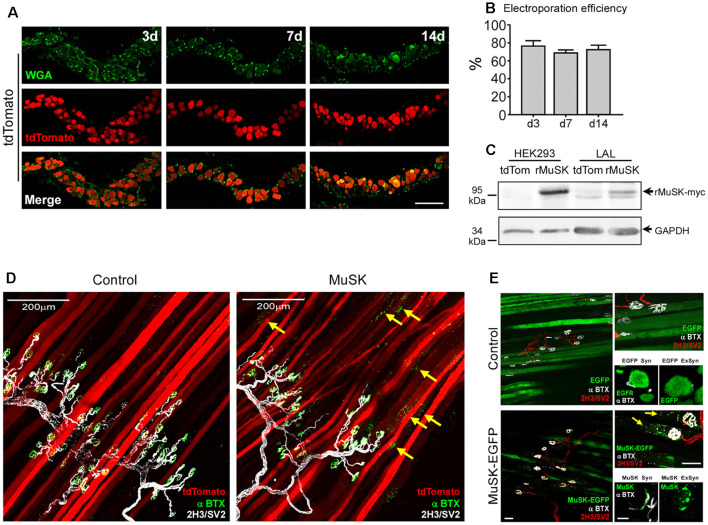
Electroporation-mediated overexpression of the MuSK receptor results in ectopic AChR aggregation. LAL muscles were transversally sectioned and stained with WGA conjugated to Alexa488 (green) to label the muscle membrane at different times after electroporation **(A)**. **(B)** Quantification of electroporation efficiency at 3, 7, and 14 days after electroporation. To analyze the efficiency of our procedure to modulate postsynaptic organization at the NMJ, LAL muscles from adult mice were co-electroporated with plasmids coding for tdTomato and rMuSK-myc to overexpress a myc-tagged version of the rat MuSK receptor and tdTomato in a 5:1 (rMuSK-myc:tdTomato) proportion. Age-matched mice subjected to tdTomato electroporation only were used as controls. **(C)** Western blot using an anti-myc antibody was performed on protein extracts from HEK293 cells and LAL muscles electroporated with tdTomato and co-electroporated with tdTomato and rMuSK-myc. After 21 days, LAL muscles were dissected and subjected to immunohistochemistry with the 2H3 (neurofilament) plus SV2 (synaptic vesicles) antibodies to reveal presynaptic motor terminals (white pseudocolor) along with Alexa488-BTX to stain postsynaptic densities **(D)**. **(E)** LAL muscles were also electroporated with the MuSK-EGFP plasmid, which contains the full-length mouse MuSK coding sequence. We used a plasmid to express EGFP as control. After 14 days, LAL muscles were dissected and subjected to immunohistochemistry with the 2H3 (neurofilament) plus SV2 (synaptic vesicles) antibodies to reveal presynaptic motor terminals (red) along with Alexa647-BTX to stain postsynaptic densities (white pseudocolor). Images at the top of the right column are magnified images of the left panels. Images at the bottom of the right column show transversal cryosections of the same LAL muscles in synaptic (Syn) and extrasynaptic (ExSyn) regions of the muscle fiber. Arrows in panels **(D,E)** show AChR clusters in extrasynaptic regions of MuSK-overexpressing LAL fibers. Scale bar 100 μm **(A)**, 200 μm **(D)**, 50 μm **(E**, *left and right top panels***)**, 10 μm (*right bottom panels*).

To further optimize our electroporation-mediated gene transfer procedure, we tried different concentrations and incubation times of hyaluronidase and combined them with varying amounts of total plasmid DNAs (data not shown). Visual inspection of the electroporation efficiency in transversal sections of LAL muscles stained with WGA at different times after electroporation (Figure 5A) showed that a 50% reduction in the time of hyaluronidase incubation (from 60–30 min) combined with a 10-fold reduction in the total DNA amount (from 80 to 8 μg) yielded high overexpression efficiencies. Quantification shows that, under these conditions, electroporation efficiency achieved an average of 77.2 ± 5.2; 69.5 ± 2.7; and 73.1 ± 4.3% (*p* > 0.05 *t*-test) of total fibers after 3, 7 and 14 days, respectively (Figure 5B). Using these optimal electroporation conditions, we conducted a functional validation of these standardized *in vivo* gene transfer conditions, which we aimed to modify the organization of the NMJ postsynaptic domain. To this aim, we used the pBK-CMV-Δlac-rMuSK plasmid (Bianchetta et al., 2005), which codes for a myc-tagged form of rat MuSK (rMuSK-myc), a muscle-specific tyrosine kinase receptor essential for NMJ assembly (DeChiara et al., 1996; Jing et al., 2009) and maintenance (Cantor et al., 2018). We also used a MuSK-EGFP plasmid, which contains the full-length mouse MuSK coding sequence fused to the EGFP protein (Ghazanfari et al., 2015; Figure 5E). We used the tdTomato plasmid as a tracer of co-electroporation with the plasmid coding for rMuSK-myc (Figures 5C,D). Control experiments confirmed the expression of rat MuSK-myc in transfected HEK293 cells and electroporated LAL muscles by Western blot using an anti-myc antibody (Figure 5C). Electroporated LAL muscles were stained to reveal postsynaptic AChR clusters and presynaptic motor axons 21 days after electroporation. Our findings show that electroporation-mediated overexpression of MuSK-myc resulted in the formation of aneural ectopic AChR clusters in most MuSK-expressing LAL muscle fibers (arrows in Figure 5D, *right panel*). Interestingly, ectopic AChR clusters distribute in discrete regions of the MuSK-overexpressing muscle fibers, thus resembling those assembled after MuSK overexpression by plasmid DNA microinjection *in vivo* (Sander et al., 2001). These AChR clusters are located hundreds of microns away from the innervation profile region (arrows in Figure 5D, *right panel*) and were completely absent in LAL muscle fibers overexpressing tdTomato only (Figure 5D, *left panel*). Similar results were obtained with the MuSK-EGFP plasmid. Transversal cryosections showed that overexpressed EGFP distributes intracellularly in synaptic and extrasynaptic regions of the muscle fibers, whereas MuSK-EGFP displayed a patched distribution along the sarcolemma and co-localizes with postsynaptic domains in synaptic regions (Figure 5E, *right bottom panels*). MuSK-EGFP overexpression also induced aneural ectopic AChR clustering in most electroporated LAL muscle fibers (Figure 5E, *left panels*). Overall, the standardization of electroporation-mediated gene transfer in the LAL muscle offers a valuable *in vivo* screening method to analyze the contribution of muscle proteins to NMJ morphology and function.

### LAL Muscle as a Model for Degenerative and Regenerative Damage to the Nerve

**Figure 6:**
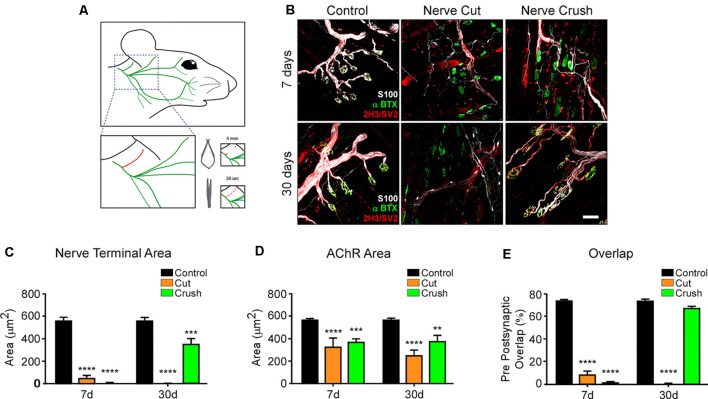
LAL muscle after degenerative and regenerative nerve damage. To study NMJ regeneration in the LAL muscle, the posterior auricular branch of the facial nerve was subjected to two protocols of injury: a portion of approximately 4 mm was transected to induce NMJ degeneration, whereas the facial nerve was crushed for 30 s to accomplish NMJ regeneration **(A)**. To analyze NMJ behavior after nerve injury, LAL muscles in the different treatments were dissected 7 (upper panels) and 30 days (lower panels) after facial nerve damage. **(B)** AChRs were labeled with α-bungarotoxin (αBTX; green), motor axons were stained with antibodies against neurofilaments, 2H3, and synaptic vesicle proteins, SV2 (red), and Schwann cells were stained with the anti S100 antibody (white). The areas of the presynaptic motor terminal **(C)** and the postsynaptic AChR-rich domain **(D)** were measured. Quantification of the apposition of postsynaptic AChR pretzels by presynaptic motor axons **(E)**. Plots represent the average ± SEM of N: 4, n: 102 (Control, black bars), N: 3, n: 116 (7 days after crush, blue bars), and N: 3, n: 105 (30 days after crush, orange bars; **p < 0.01; ***p < 0.001, ****p < 0.0001, one-way ANOVA). Scale bar 25 μm **(B)**.

One main condition to test the role of muscle-derived proteins is the process of NMJ regeneration. Therefore, as a first hint to explore the potential use of the LAL muscle as a model of NMJ regeneration, we refined a previously described procedure to accomplish facial nerve axotomy (Olmstead et al., 2015) and analyzed NMJ degeneration and regeneration. With this aim, we performed two procedures of nerve damage; to analyze NMJ degeneration, a 4 mm segment of the posterior auricular branch of the facial nerve was transected, whereas NMJ regeneration was evaluated after a 30 s crush injury of the same facial nerve branch (Figure 6A). LAL muscles were stained to reveal postsynaptic AChR clusters, presynaptic motor axons, and Schwann cells 7 and 30 days after nerve damage (Figure 6B). In both procedures, motor axons showed degeneration 7 days after facial nerve injury, leading to the denervation of the NMJ postsynaptic domains (Figure 6B). Also, terminal Schwann cells extended long projections away from the synaptic domains, as previously described in hind limb muscles (Reynolds and Woolf, 1992). Thirty days after facial nerve resection, postsynaptic AChR clusters suffered a transition from branched pretzel-like mature shapes to fragmented and blurry ones, whereas terminal Schwann cells still display long projections (Figure 6B). In turn, within a similar time frame after facial nerve crush, motor axons have regrown re-innervating the pretzel-like postsynaptic domains, whereas terminal Schwann cells retracted their projections (Figure 6B). Quantification of synaptic parameters shows a strong reduction of the nerve terminal area 7 days after both procedures of nerve injury. This value is recovered 30 days after nerve crush, although it does not reach control values (Figure 6C). Similarly, the area of postsynaptic AChR clusters is also decreased to a similar extent 7 and 30 days after facial nerve cut and crush (Figure 6D). As a consequence, the overlap of pre/postsynaptic apposition decreases immediately after injury and returns to basal levels 30 days post nerve crush injury (Figure 6E). Even though the pre/postsynaptic apposition is significantly lower 30 days after injury, the overlap value (68.37 ± 1.351%) fits with complete NMJ reinnervation (Jones et al., 2016). As expected, pre/postsynaptic apposition does not exhibit any recovery 30 days after nerve cut (Figure 6E). Along with our previous results, the standardization of these two models of facial nerve injury allows for the study of proteins participating in the process of NMJ regeneration in the LAL muscle.

## Discussion

Several pathological conditions negatively affect NMJ integrity as a primary target. Compared to the central nervous system, the peripheral nervous system bears a higher regenerative ability; however, this process only occurs under permissive environmental conditions for a successful repair. One such crucial step is the stabilization of denervated muscle postsynaptic domains until they become re-innervated. Even though pretzel-like AChR clusters maintain their gross shape for several weeks after injury (Akaaboune et al., 1999), detailed analyses have demonstrated injury-dependent loss and gain of AChRs in entire pretzel branches within postsynaptic regions, an effect that increases at longer denervation times (Kang et al., 2014). In this context, seeking for suitable models to analyze the contribution of muscle-derived molecules on postsynaptic NMJ stability for re-assembly is a permanent need. Our procedure constitutes a rapid, easily reproducible, and reliable screening method to modify the expression of candidate muscle-derived proteins in a time-dependent manner and to analyze their potential effect on NMJ behavior. In this regard, the LAL muscle offers unique experimental advantages for NMJ studies (Angaut-Petit et al., 1987; Erzen et al., 2000; Wright et al., 2011; Burke et al., 2018). It is a superficially and easily accessible muscle, which allows repeated *in vivo* manipulation and visualization, it is a flat and thin muscle, which facilitates the “*en face*” observation of NMJs in the optical plane of the microscopes in whole-mount preparations, and it allows nerve/muscle preparations for electrophysiological recording (Katz et al., 1996; Ruiz et al., 2010). Our studies also reveal the usefulness of this approach to analyze presynaptic morphology and apposition as well as the dynamic processes commanding NMJ maturation, including the neurotransmitter release dynamics and the consolidation of postsynaptic morphologies. Our findings demonstrate that the electroporation and the persistent expression of a control fluorescent protein in LAL muscle fibers during the transition from immature to mature NMJs do not modify neurotransmission parameters, further supporting the benefits of this procedure to characterize the effect of muscle-derived proteins on NMJ function.

How do muscle-derived proteins contribute to the stability of mature NMJs? Even though the original view stated dominant roles for motor neurons in NMJ formation, an increasing amount of evidence has highlighted the key contribution of skeletal muscle processes for embryonic NMJ assembly. For instance, an aneurally induced pre-pattern of AChR clusters guide motor axons for proper NMJ installation (Jing et al., 2009). Cumulative evidence also suggests that muscle proteins that act as main regulators of embryonic NMJ assembly are required for mature NMJ maintenance. These include proteins involved in the signaling triggered by agrin, a major motor neuron-derived organizer of the NMJ, such as the MuSK receptor (Bowen et al., 1998), its LRP4 co-receptor (Weatherbee et al., 2006), and the intracellular effectors Dok-7 (Okada et al., 2006), and rapsyn (Bruneau and Akaaboune, 2010). Auto-antibodies against MuSK and LRP4 cause Myasthenia gravis (Verschuuren et al., 2013; Plomp et al., 2015), whereas mutations affecting the function of each of these proteins are involved in Myasthenic Congenital Syndromes (Engel et al., 2015), thus demonstrating the requirement of these postsynaptic proteins for mature NMJ maintenance. In this regard, *in vivo* gene transfer approaches have been efficiently employed to analyze the effect of muscle proteins on NMJ behavior (Kong et al., 2004; Sadasivam et al., 2005; Martínez-Martínez et al., 2007; Bruneau and Akaaboune, 2010; Punga et al., 2011; Chen et al., 2014; Gomez et al., 2016; Wang et al., 2017). For instance, electroporation-mediated modulation of rapsyn or Dok7 has allowed studies of its distribution at the mature NMJ and its effect on muscle response (Martínez-Martínez et al., 2007, 2009; Bruneau and Akaaboune, 2010; Gomez et al., 2016). Also, electrotransfer of a dsRNA sequence to silence MuSK expression in the *Soleus* muscle resulted in postsynaptic disruption (Kong et al., 2004). Interestingly, previous studies had shown that MuSK overexpression by plasmid microinjection in individual *Soleus* muscle fibers induced the formation of ectopic AChR clusters (Sander et al., 2001). By using electroporation-mediated gene transfer in LAL muscle fibers, here we have recapitulated these results by showing that most MuSK-expressing fibers display ectopic AChR clusters.

A key level for potential therapeutic interventions aimed at NMJ regeneration is to identify molecules and mechanisms that help AChR stability after denervation. Our study extends the usefulness of the LAL muscle to analyze NMJ regeneration. Following a facial nerve crush injury protocol, we observed axonal degeneration and muscle fiber reinnervation similar to that described in the hind limb *Tibialis anterior* muscle after sciatic nerve crush injury (Magill et al., 2007). In the context of NMJ regeneration, several muscle proteins that play essential roles in the embryonic assembly have demonstrated a potentially key role on helping NMJ maintenance, such as LRP4 (Barik et al., 2014), Dok-7 (Eguchi et al., 2016), and rapsyn (Kong et al., 2004; Martínez-Martínez et al., 2009). Indeed, overexpression of a low copy number of MuSK partially rescues the NMJ degeneration that takes place at pre-symptomatic stages of Amyotrophic Lateral Sclerosis animal models (Pérez-Garcia and Burden, 2012). Remarkably, these studies led to the discovery that antibody-mediated activation of endogenous MuSK has the same rescue effect (Cantor et al., 2018), opening a therapeutic alternative to prevent NMJ alterations in this and other pathological conditions resulting in neuromuscular synapse damage. It is also relevant to mention that successful NMJ regeneration is helped by signals derived from motor axon terminals and Schwann cells. For instance, nerve-released neurotrophic factors act as essential molecules to promote functional NMJ reinnervation (Bendella et al., 2018), whereas terminal Schwann cell-derived guidance molecules, such as CXCL12α, play essential roles in guiding motor axons to muscle fibers for functional repair (Negro et al., 2017). Also, recent findings reveal that local delivery of VEGF plus IGF-1, specifically at the distal site of sciatic nerve injury (i.e towards the NMJ), promoted functional reinnervation and muscle regeneration (Raimondo et al., 2019). These findings reveal that, regardless of the cellular origin of relevant trans-synaptic secreted proteins, electroporation of skeletal muscles as a common *in vivo* source of these molecules is a relevant therapeutic strategy to accomplish local beneficial effects on NMJ regeneration.

Therefore, in our view, protocols combining facial nerve injury and *in vivo* LAL muscle electroporation-mediated gene delivery are a reliable and fast strategy to investigate the potential role of muscle proteins on NMJ structure, function, and regeneration. Also, these methods can be used as a screening procedure before facing more complex and long-lasting approaches such as the generation of transgenic animal models.

## Data Availability Statement

The raw data supporting the conclusions of this article will be made available by the authors, without undue reservation.

## Ethics Statement

The animal study was reviewed and approved by Bioethics Committee, Universidad de Concepción, Concepción, Chile.

## Author Contributions

JO, FB-G, LT, and JH contributed to the conception and design of the work. JO, FB-G, VP, JM, PH, DH, RT, and ML-M contributed experiments and data collection. JO, FB-G, LT, and JH contributed data analysis and interpretation. JO, LT, and JH wrote the manuscript. All authors contributed to the critical revision of the article and approve its final submitted version.

## Conflict of Interest

The authors declare that the research was conducted in the absence of any commercial or financial relationships that could be construed as a potential conflict of interest.
